# Identification of chloride intracellular channels as prognostic factors correlated with immune infiltration in hepatocellular carcinoma using bioinformatics analysis

**DOI:** 10.1097/MD.0000000000027739

**Published:** 2021-11-12

**Authors:** Juan-Jun Huang, Jing Lin, Xiaoli Chen, Wei Zhu

**Affiliations:** a Department of Infectious Diseases, The Affiliated Ganzhou Hospital of Nanchang University, Ganzhou, Jiangxi, PR China; b Central Laboratory, The Affiliated Ganzhou Hospital of Nanchang University, Ganzhou, Jiangxi, PR China.

**Keywords:** bioinformatics analysis, chloride intracellular channel, hepatocellular carcinoma, immune infiltration, prognosis

## Abstract

Chloride intracellular channel (CLIC) proteins are novel Cl-channels with 6 family members (CLIC1–6) that are known to play crucial roles in multiple physiological functions, such as neurological, cardiovascular, pulmonary, and auditory functions, and in various malignancies, including hepatocellular carcinoma (HCC). However, considerable challenges exist in identifying appropriate CLICs as therapeutic target molecules and prognostic biomarkers for HCC because the transformation of soluble or integral membrane protein forms, and specific pharmacological agents (agonists and antagonists) for distinct CLICs remains enigmatic.

To address this issue and the possible molecular basis and the signaling networks activated by CLICs in HCC, we examined the transcriptional, promoter methylation, DNA mutation, survival, and immune infiltration data of CLICs in patients with HCC using the ONCOMINE, UALCAN, GEPIA, cBioPortal, and TIMER databases.

The data showed that the expression levels of CLIC family members were differed between tumor and normal tissues. High expression levels of CLIC1 and CLIC3 were associated with advanced cancer stage in HCC patients. Low CLIC1 expression was associated with a better overall survival (OS). The DNA methylation levels of the CLIC1–3 and CLIC5–6 promoters in tumor tissue with HCC were significantly lower in HCC tissues than in normal tissues. Patients with CLIC1 alterations had a shorter OS than patients with unaltered CLIC1. Moreover, the expression levels of CLICs correlated with the infiltration of 6 different immune cells (B cells, CD4^+^ T cells, CD8^+^ T cells, neutrophils, macrophages, and dendritic cells).

These results indicate that the increased mRNA expression and decreased promoter DNA methylation level of CLICs may play crucial roles in HCC tumorigenesis. The expression of CLIC family members was significantly correlated with the tumor immune status. High CLIC1 and CLIC3 expression levels could serve as biomarkers for identifying advanced-stage HCC. Moreover, a CLIC1 mutation rate of 18% was also observed and CLIC1 genetic alterations were significantly associated with lower OS in HCC patients.

## Abstract 2

Supplemental Digital Content is available in the text

## 1 Introduction

Hepatocellular carcinoma (HCC) comprise 75% to 85% of all primary carcinomas of the liver. It was the sixth most common cancer (fifth most common in men) and the fourth most common cause of death from cancer worldwide (second in men) in 2018. It is expected that HCC will be responsible for 1.35 million new cancer cases and 1.28 million cancer-related deaths by 2040.^[1,2]^

In most patients, HCC is diagnosed in the advanced stages, with an expected survival time of <5 years. This is due to the limited symptoms and available biomarkers in the early stages of HCC and a dismal 3-year survival rate of 12.7%.^[3]^

Ion channels are considered highly effective pharmacological targets because ion homeostasis is associated with various important biological processes, such as oxidative stress, apoptosis, autophagy, and inflammation.^[4]^ Chloride intracellular channel (CLIC) proteins are novel Cl channels with 6 mammalian paralogs.^[5,6]^ They exist in tow forms (soluble proteins, and integral membrane proteins), and act as enzymes and channels.^[7]^ CLIC proteins can perform cellular functions by serving as cytoplasmic reagents or membrane ionic transduction materials owing to their polymorphism.^[8]^ Many studies have shown that the CLIC family may be useful in regulating some important physiological and pathological functions, including cardiovascular and pulmonary functions; may be implicated in hearing impairment and neurophysiology diseases; and may especially be important in regulating cell growth and apoptosis for novel biomarkers.^[9]^

Previous studies have made considerable progress in elucidating the molecular identity, localization, and biophysical properties of CLICs. They demonstrated that CLICs play a crucial role in neurological, cardiovascular, pulmonary, and auditory functions, as well as in various malignancies, including HCC. Many studies related to CLICs in HCC have been conducted to date; however, they mainly focused on CLIC1. Studies related to epigenetics and the immune microenvironment are lacking. The transformation of soluble or integral membrane protein forms and specific pharmacological agents (agonists and antagonists) for distinct CLICs is still not well understood.^[7]^ Therefore, considerable challenges remain in identifying appropriate CLICs as therapeutic target molecules and prognostic biomarkers for HCC. The rapid growth of gene sequencing technology and bioinformatics databases has enabled the complete analysis of CLICs. In this study, we examined large public databases and conducted a bioinformatics analysis of the transcriptional expression, gene mutation, and promoter methylation of CLICs, as well as analyzed the immune cell infiltration in HCC to assess the potential of CLICs as target molecules and prognostic biomarkers.

## 2 Materials and methods

### 2.1 ONCOMINE

ONCOMINE (www.oncomine.org) collects microarray data of mRNA expression or DNA copy number in primary tumors, cell lines, or xenografts, usually from published studies. The database can be used to analyze cancer and normal tissues.^[10]^ In this study, data were collected to analyze and compare the mRNA levels of the 6 different members of CLIC between HCC and normal tissues. Student *t* test was used, and *P* values, fold change, and gene rank were set at.05, 1.5, and 10%, respectively.

### 2.2 UALCAN

UALCAN (http://ualcan.path.uab.edu/analysis.html) uses The Cancer Genome Atlas (TCGA) data of 31 types of cancer for in-depth analyses of gene expression data.^[11]^ We analyzed the mRNA expression of the 6 CLIC members in tumor and normal tissues using UALCAN. Further, we evaluated the epigenetic regulation of promoter methylation and gene expression and their relevance. Statistical significance was defined as *P* < .05.

### 2.3 GEPIA

GEPIA (http://gepia2.cancer-pku.cn/#analysis) is a portal with a standard process for analyzing the RNA-seq data of 9736 cancer cases and 8587 normal cases from TCGA and Genotype Tissue Expression programs.^[12]^ GEPIA provides differential expression analysis of the collection between tumor and normal tissues according to the 6 different members of CLICs and the pathological stage of the disease through one-way analysis of variance. We analyzed the overall survival (OS) of HCC patients between the high expression group and the low expression group, with automatic selection of the best mRNA expression cutoff values for the 6 members of CLICs based on RNA-seq and assessments using Kaplan-Meier survival curves. Statistical significance was defined as a *P* value < .05.

### 2.4 cBioPortal

cBioPortal (www.cbioportal.org) is a portal used by researchers to explore, visualize, and analyze the multidimensional cancer genomics database in TCGA.^[13]^ We analyzed the genomic profiles of the 6 CLIC family members, with all 3 types of genomic profiles (z-score threshold = ±1.5). The associations between the genetic mutations of CLICs and OS were displayed as Kaplan-Meier plots. Statistical significance was defined as a *P* value < .05.

### 2.5 GeneMANIA

GeneMANIA (https://genemania.org/) is an easy-to-use portal for finding the related genes of a series of target genes, using a large amount of functional association data including protein and genetic interactions, pathways, co-expression, co-localization, and protein domain similarity.^[14]^ In this study, we performed a network analysis of the 6 CLIC members and their 20 functionally related genes.

### 2.6 WebGestalt

WebGestalt (http://webgestalt.org) is a web page that is extensively used for interpreting the gene lists of interest using large-scale omics data and functional enrichment analysis.^[15–18]^ This study performed a network analysis of the 6 CLIC members and their 20 functionally related genes. Moreover, functional enrichment of CLIC members and their 20 functionally related genes was analyzed using Gene Ontology (GO) analysis and Kyoto Encyclopedia of Genes and Genomes (KEGG) pathway analysis. The Run program is the “WebGestalt” R package. Statistical significance was defined as a *P* value < .05. GO analysis includes biological process (BP), cellular component (CC), and molecular function categories.

### 2.7 TIMER

TIMER (https://cistrome.shinyapps.io/timer/) is a web with integrated resources for systematically analyzing immune infiltrates, including CD4^+^ T cells, CD8^+^ T cells, B cells, macrophages, neutrophils, and dendritic cells (DCs) across different types of cancer.^[19,20]^ We used this tool for the correlation analysis between the expression of the 6 distinct CLIC members and patient survival and infiltration of immune cells in HCC. Statistical significance was defined as a *P* value < .05.

## 3 Results

### 3.1 Differential expression of distinct CLICs members in HCC patients

**Figure 1:**
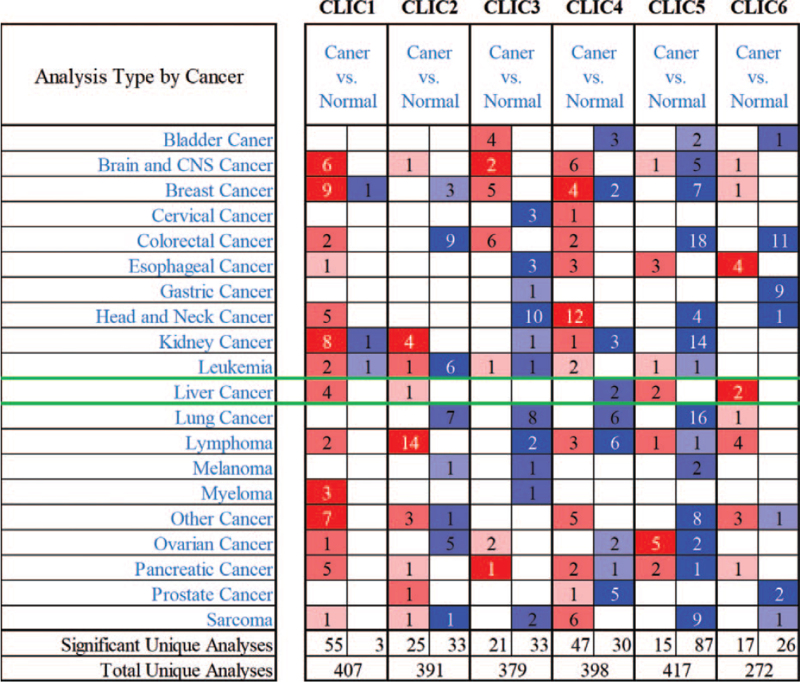
Transcriptional expression of CLICs in 20 different types of cancer diseases (ONCOMINE). Differences in transcriptional expression were compared using Student *t* test. Cut-off of *P* value and fold change were as follows: *P* value:.01, fold change: 1.5, gene rank: 10%, data type: mRNA. CLIC = chloride intracellular channel, CNS = central nervous system.

**Figure 2:**
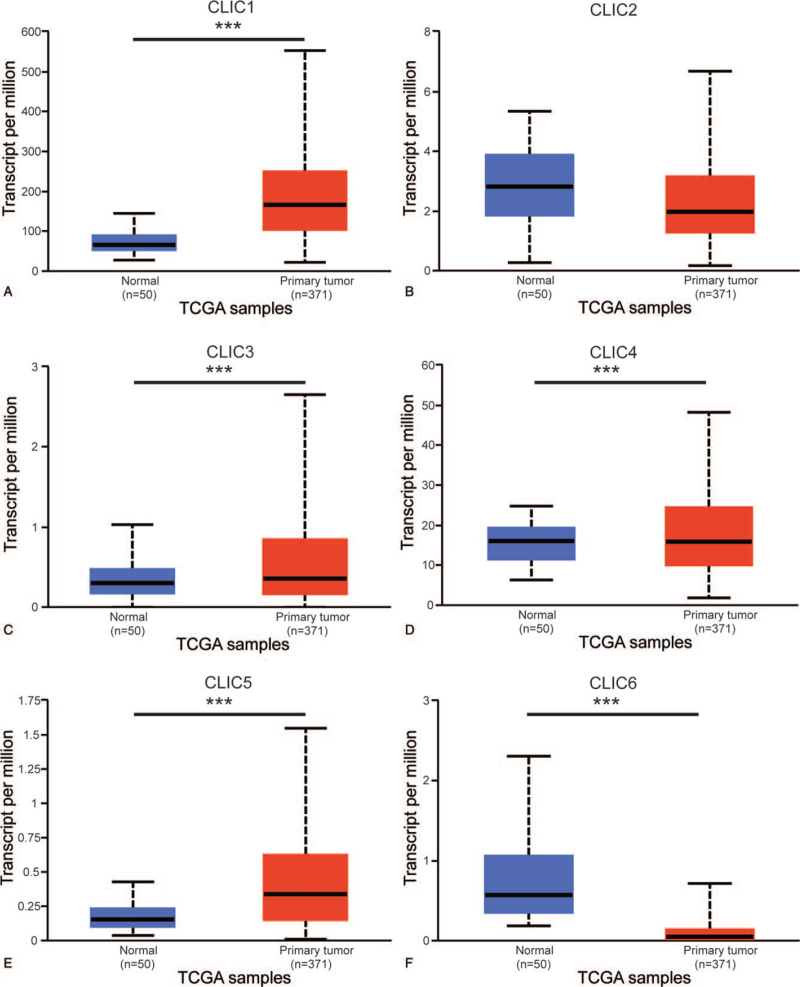
mRNA expression of distinct CLICs family members in HCC tissues and adjacent normal liver tissues (UALCAN). CLIC = chloride intracellular channel, HCC = hepatocellular carcinoma, TCGA = The Cancer Genome Atlas, ^∗∗∗^*P* < .001.

To investigate the different prognostic and therapeutic values of different CLIC members in HCC patients, we analyzed the expression of mRNA using the ONCOMINE and UALCAN databases. Figure 1 and Table 1 show the differences in the mRNA expression of 6 CLIC family members in 20 types of cancer and normal tissues in ONCOMINE. Significantly higher mRNA expression of CLIC1 and CLIC5 was found in the HCC samples in multiple datasets. The Mas Liver dataset showed CLIC1 overexpression in HCC tissues, with approximately 838 fold change (*P* = 6.84E-11) compared with normal tissues.^[21]^ Roessler Liver 2 dataset demonstrated a 2.371-fold increase in CLIC1 mRNA expression in HCC samples (*P* = 3.33E-61)^[22]^ and Roessler Liver observed a 2.655-fold increase in CLIC1 mRNA expression in HCC tissues (*P* = 2.86E-07).^[22]^ The results from the Wurmbach Liver dataset showed that there was a 1.606-fold (*P* = 4.87E-05) increase in CLIC5 mRNA expression in HCC tissues.^[23]^ We used UALCAN to further analyze the mRNA expression levels of 6 CLIC family members based on the TCGA database, which differs from the ONCOMINE database. Figure 2 shows the mRNA expression levels of 4 CLIC family members (CLIC1, CLIC3, CLIC4, and CLIC5), which were significantly upregulated in HCC tissues compared with normal tissues *(all P* < .001). CLIC6 was significantly downregulated in HCC tissues compared with the level in normal tissues.

**Table 1:** Significant changes of CLICs expression in transcription level between HCC and normal liver tissues (ONCOMINE).

CLICs member	Types of cancer vs liver	Fold change	*P* value	*t* test	Source and reference
CLIC1					
	Hepatocellular carcinoma vs normal	1.838	6.84E-11	7.933	Mas Liver^[21]^
	Hepatocellular carcinoma vs normal	2.371	3.33E-61	19.643	Roessler Liver 2^[22]^
	Hepatocellular carcinoma vs normal	2.655	2.86E-07	6.361	Roessler Liver^[22]^
CLIC5					
	Hepatocellular carcinoma vs normal	1.606	4.87E-05	4.351	Wurmbach Liver^[23]^

CLIC = chloride intracellular channel, HCC = hepatocellular carcinoma.

### 3.2 Association of mRNA expression of different CLIC family members with tumor stage

**Figure 3:**
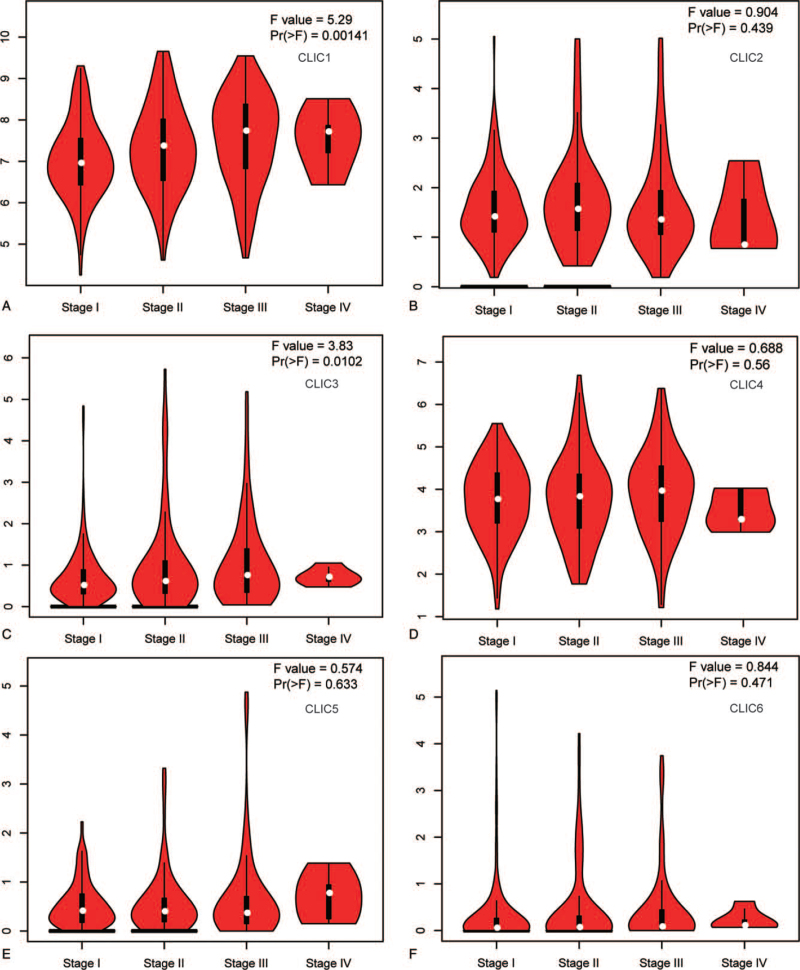
Relationship between the mRNA expression of distinct CLICs family members and individual cancer stages of HCC patients (GEPIA). CLIC = chloride intracellular channel, HCC = hepatocellular carcinoma.

The relationship between the mRNA levels of different CLIC family members and the cancer stages of HCC patients was analyzed using GEPIA. Figure 3 shows that the mRNA expression of CLIC1 and CLIC3 was significantly correlated with the pathological stage, and the expression of mRNA in CLICs was higher in patients with advanced cancer.

### 3.3 Association of the CLIC mRNA expression with OS in HCC patients

**Figure 4:**
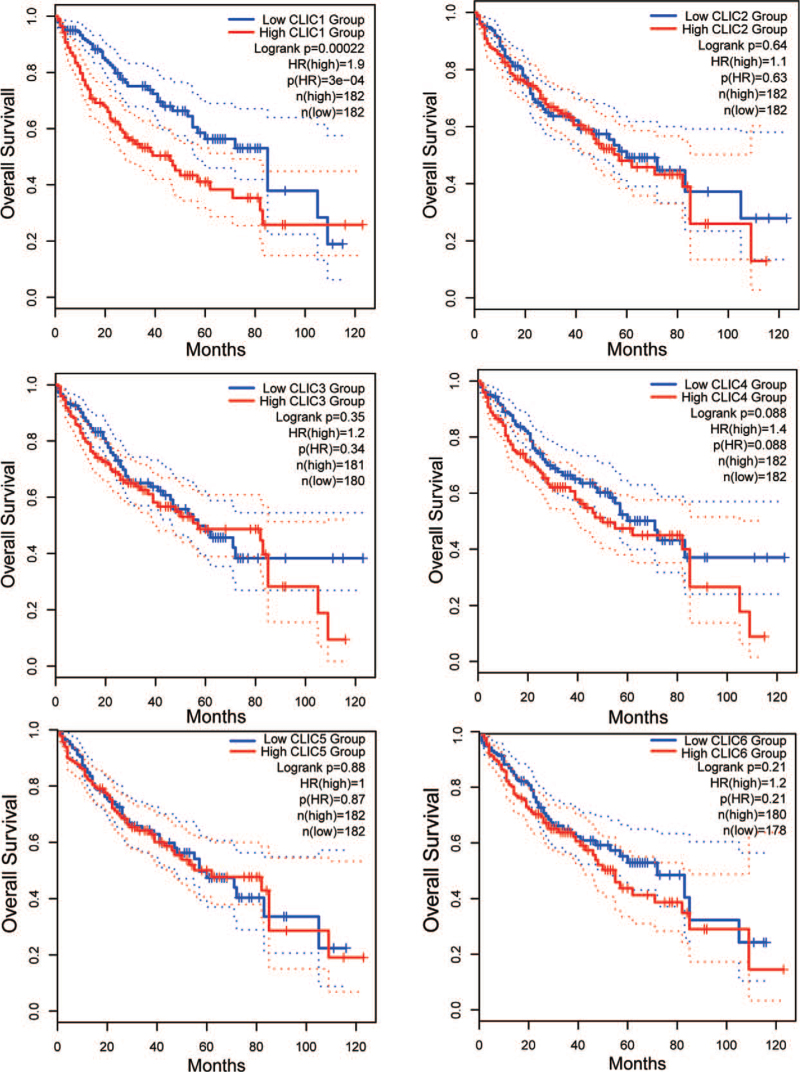
Prognostic value of the mRNA expression of distinct CLICs family members in HCC (GEPIA). CLIC = chloride intracellular channel, HCC = hepatocellular carcinoma, HR = hazard ratio.

GEPIA was used to further analyze the correlation between the mRNA levels of CLICs and the OS in HCC patients. The analysis revealed that increased CLIC1 mRNA levels were significantly associated with lower OS (*P* < .01) in HCC patients. No significant differences were observed for the other 5 CLIC members (Fig. 4).

### 3.4 Network and functional enrichment analysis of CLICs and functionally related genes in HCC

**Figure 5:**
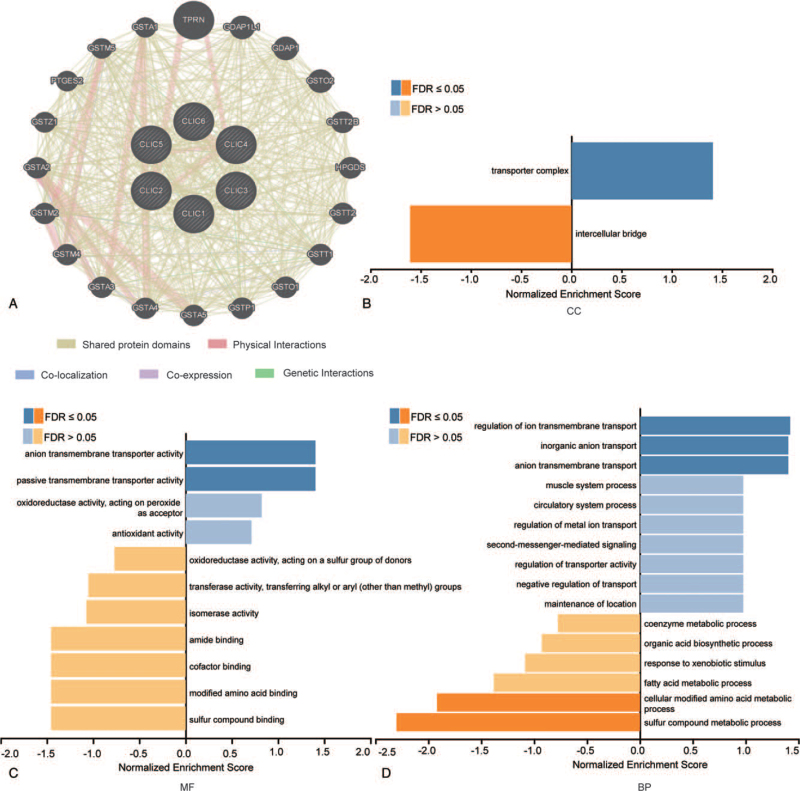
Predicted functions and pathways of the mutations in CLICs and 20 functional neighboring genes in HCC (GeneMANIA and WebGestalt). Network of CLICs and their 20 function neighbor genes was constructed (A), GO functional enrichment analysis predicted 3 main functions of CLICs and their 20 function neighbor genes,(B) CC, (C) MF, and (D) BP, BP = biological processes, CC = cellular components, CLIC = chloride intracellular channel, FDR = false discovery rate, GDAP1 = ganglioside induced differentiation associated protein 1, GDAP1L1 = ganglioside induced differentiation associated protein 1-like 1, GSTA1 = glutathione S-transferase alpha 1, GSTA2 = glutathione S-transferase alpha 2, GSTA3 = glutathione S-transferase alpha 3, GSTA4 = glutathione S-transferase alpha 4, GSTA5 = glutathione S-transferase alpha 5, GSTM2 = glutathione S-transferase mu 2, GSTM4 = glutathione S-transferase mu 4, GSTM5 = glutathione S-transferase mu 5, GSTO1 = glutathione S-transferase omega 1, GSTO2 = glutathione S-transferase omega 2, GSTP1 = glutathione S-transferase pi 1, GSTT1 = glutathione S-transferase theta 1, GSTT2 = glutathione S-transferase theta 2, GSTT2B = glutathione S-transferase theta 2B, GSTZ1 = glutathione S-transferase zeta 1, HCC = hepatocellular carcinoma, HPGDS = hematopoietic prostaglandin D synthase, MF = molecular function, PTGES2 = prostaglandin E synthase 2, TPRN = taperin.

Using GeneMANIA, we performed a network analysis of 6 CLIC members and their 20 functionally related genes, as follows: taperin, ganglioside induced differentiation associated protein 1-like 1, ganglioside induced differentiation associated protein 1, glutathione S-transferase omega 2, glutathione S-transferase theta 2B, hematopoietic prostaglandin D synthase, glutathione S-transferase theta 2, glutathione S-transferase theta 1, glutathione S-transferase omega 1, glutathione S-transferase pi 1, glutathione S-transferase alpha 5, glutathione S-transferase alpha 4, glutathione S-transferase alpha 3, glutathione S-transferase mu 4, glutathione S-transferase mu 2, glutathione S-transferase alpha 2, glutathione S-transferase zeta 1, prostaglandin E synthase 2, glutathione S-transferase mu 5, glutathione S-transferase alpha 1. All CLICs share protein domains: CLIC1, CLIC4, CLIC5, and CLIC6 show genetic interactions with each other; CLIC2, CLIC4, CLIC5 and CLIC6 form physical interactions with each other; CLIC1 and CLIC3 are coexpressed molecules; and CLIC1and CLIC2 colocalize within cells (Fig. 5A). The functions and KEGG pathway enrichment of CLICs and their 20 neighbor genes were analyzed using WebGestalt. Functional enrichment included 3 groups: CC group (2 terms), BP group (16 terms), and molecular function group (11 terms) (Fig. 5 B-D and Table 2). With respect to BP, GO:0006575 cellular modified amino acid metabolic process, GO:0006790 sulfur compound metabolic process, GO:0034765 regulation of ion transmembrane transport, GO:0015698 inorganic anion transport, and GO:0098656 anion transmembrane transport were remarkably regulated by CLICs and 20 neighbor genes. For CC, GO:1990351 transporter complex, GO:0045171 intercellular bridge was significantly associated with them. In addition, GO:0008509 anion transmembrane transporter activity and GO:0022803 passive transmembrane transporter activity also affected the molecular functions. KEGG pathway enrichment indicated that hsa00983 drug metabolism, hsa01524 platinum drug resistance, hsa05200 Pathways in cancer, hsa05225 hepatocellular carcinoma, hsa05418 fluid shear stress and atherosclerosis, hsa00480 glutathione metabolism, hsa00980 metabolism of xenobiotics by cytochrome P450, hsa00982 drug metabolism, and hsa05204 chemical carcinogenesis were associated with the functions of CLICs and neighboring genes (Table 2).

**Table 2:** Significantly enriched GO terms and KEGG pathways of CLICs and neighbor genes in HCC (WebGestalt).

Term	Description	Count	FDR
GO:1990351	Transporter complex	6	0.00000
GO:0045171	Intercellular bridge	3	0.02703
GO:0006790	Sulfur compound metabolic process	16	0.00000
GO:0034765	Regulation of ion transmembrane transport	8	0.00092
GO:0006575	Cellular modified amino acid metabolic process	13	0.00189
GO:0015698	Inorganic anion transport	6	0.00245
GO:0098656	Anion transmembrane transport	6	0.00245
GO:0008509	Anion transmembrane transporter activity	6	0.00046
GO:0022803	Passive transmembrane transporter activity	6	0.00046
hsa00480	Glutathione metabolism	15	0.00000
hsa00980	Metabolism of xenobiotics by cytochrome P450	15	0.00000
hsa05204	Chemical carcinogenesis	15	0.00000
hsa00983	Drug metabolism	14	0.00432
hsa01524	Platinum drug resistance	14	0.00432
hsa05200	Pathways in cancer	14	0.00432
hsa05225	Hepatocellular carcinoma	14	0.00432
hsa05418	Fluid shear stress and atherosclerosis	14	0.00432
hsa00982	Drug metabolism	15	0.00432

CLIC = chloride intracellular channel, FDR = false discovery rate, GO = Gene Ontology, HCC = hepatocellular carcinoma, KEGG = Kyoto Encyclopedia of Genes and Genomes.

### 3.5 Genetic alterations of CLICs in HCC

**Figure 6:**
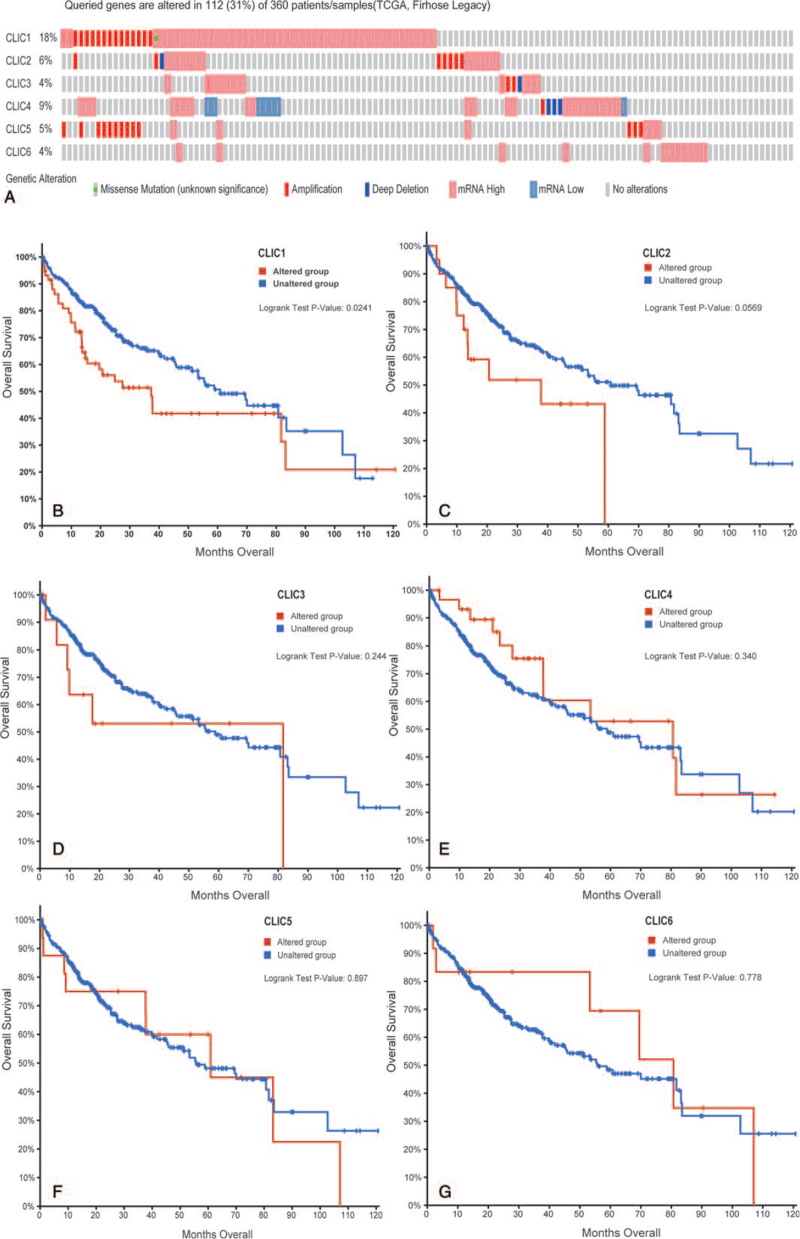
Alteration frequencies of CLIC members and their association with OS in HCC (cBioPortal). (A) Mutation rates of 6 CLIC members. (B-G) Comparing OS in HCC patients with/without CLIC mutation, CLIC = chloride intracellular channel, HCC = hepatocellular carcinoma, TCGA = The Cancer Genome Atlas.

We used the cBioPortal dataset to investigate the value of CLIC genetic alterations in HCC patients. There were 5 types of gene alterations, amplifications, deep deletions, mRNA high expression, mRNA low expression, and multiple alterations (Fig. 6A). Of the 360 patients, 112 had genetic alterations (31%). The percentages of genetic alterations in CLIC1–6 were 18%, 6%, 4%, 9%, 5%, and 4%, respectively. Our results indicated that the alterations of CLIC1 had a significant correlation with lower OS in HCC patients (*P* = .00241) (Fig. 6B-G), whereas there were no significant correlations for the other 5 members.

### 3.6 Association of expression of CLIC with promoter DNA methylation and immune cell infiltration

**Figure 7:**
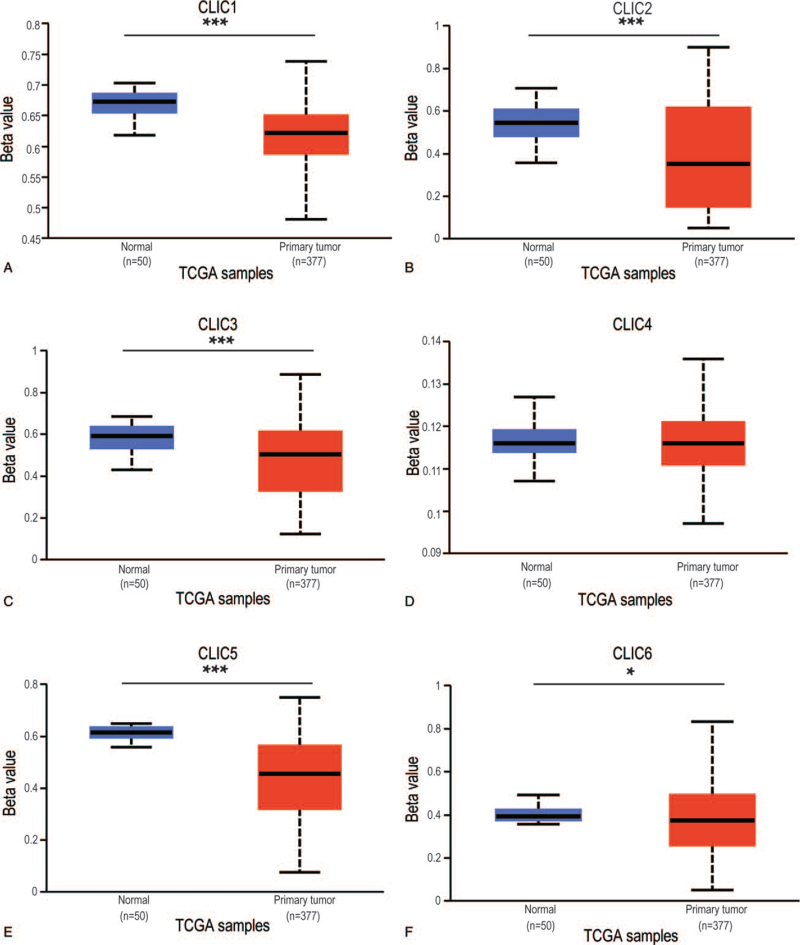
The Beta value indicates the level of promoter methylation of distinct CLICs family members in HCC tissues and para-carcinoma tissues (UALCAN). CLIC = chloride intracellular channel, HCC = hepatocellular carcinoma, TCGA = The Cancer Genome Atlas, ^∗∗∗^*P* < .001, ^∗^*P* < .05.

**Figure 8:**
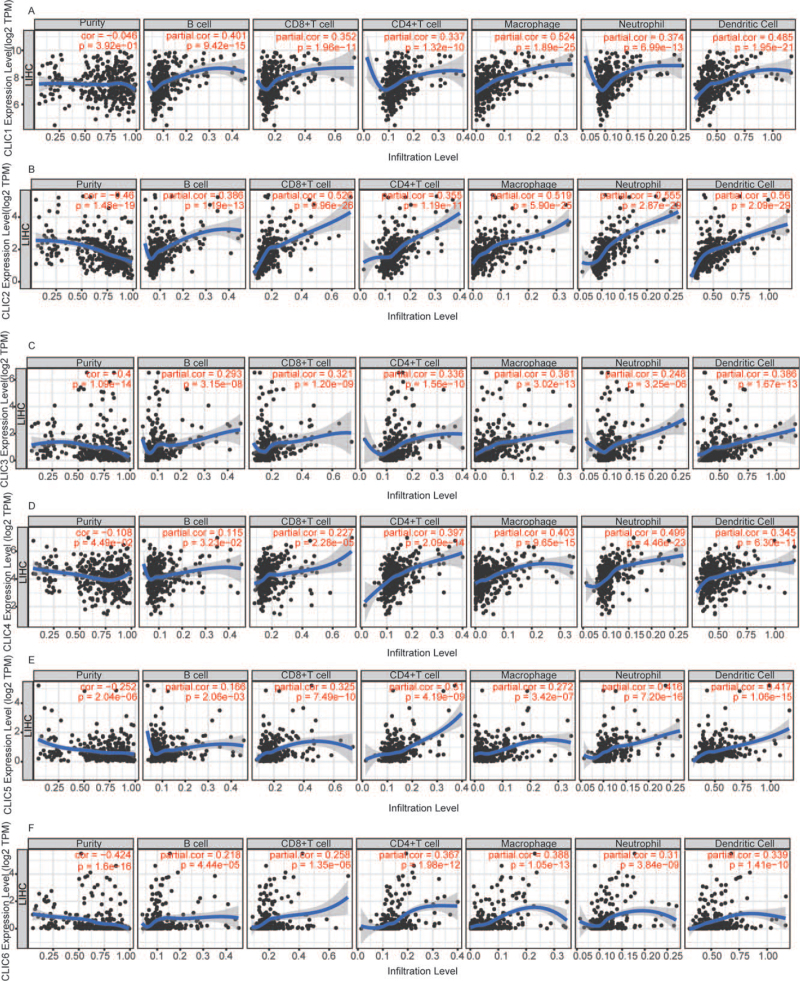
The correlation between distinct CLICs members and immune cell infiltration (TIMER). CLIC = chloride intracellular channel, LIHC = liver hepatocellular carcinoma.

To provide novel clues that may reveal the possible molecular mechanism at the epigenetic level, we examined the correlations of the 6 distinctly expressed CLICs, with promoter methylation, and immune infiltration. The DNA methylation levels of CLIC1–3 (*P* < .001), CLIC5 (*P* < .001), and CLIC6 (*P* < .05) promoters were significantly lower in HCC tissues than in normal tissues (Fig. 7A-F). The data showed that the expression of all CLICs was significantly positively correlated with 6 types of infiltrating immune cells (B cells, CD8^+^ T cells, CD4^+^ T cells, neutrophils, macrophages, and DCs) *(P* < .005) (Fig. 8). Moreover, the Kaplan-Meier (KM) curves showed that the prognosis of patients with CLIC1-low, CLIC2-high, CLIC4-low, CLIC5-low, and CLIC6-high expression was poor in the subgroup with high levels of neutrophil infiltration (Figure S1A,B,E,G,I, Supplemental Digital Content, http://links.lww.com/MD/G476). Patients with CLIC2-high, CLIC4-high, and CLIC5-low expression had worse OS in subgroup with the high infiltration of macrophages (Figure S1C,F,H, Supplemental Digital Content, http://links.lww.com/MD/G476). Furthermore, the infiltration of DCs was negatively correlated with patients with CLIC3-low expression (Figure S1D, Supplemental Digital Content, http://links.lww.com/MD/G476).

## 4 Discussion

To date, many studies related to CLICs in HCC have been published; however, they mainly focused on CLIC1. Previous studies have shown that CLIC1 is upregulated in HCC tissues and cell lines,^[24–31]^ promotes the invasion and migration of HCC cells in vivo and in vitro,^[25,27–30,32]^ and is a predictor of worse survival.^[27,30]^ CLIC1 promotes HCC migration and invasion by decreasing the expression of maspin, annexin A7, and gelsolin, which could be inhibited by miR-124, and miR-122-5p overexpression, or TP53 depletion.^[28–30,32]^ With respect to the other CLIC members, the CLIC2 mRNA expression level was found to be significantly lower in HCC tissues, which may be related to the lack of tight junctions that lead to metastasis of HCC.^[31]^ CLIC3 was upregulated in HCC cells and was associated with a short OS and improved tumor cell migration and metastasis by regulating membrane ruffling, and may act as a novel target of epigallocatechin gallate and fisetin.^[33,34]^ Flores-Tellez et al^[35]^ showed that CLIC5 is upregulated in rat liver tumors, promoting tumor cell proliferation, migration potential, and invasion capacity, and was co-localized with podocalyxin, and CLIC5 in tumor regions.

HCC develops owing to the abnormal regulation of multiple genetic, epigenetic, and signaling pathways that interact with the tumor microenvironment, promoting the occurrence, development, and metastasis of tumors.^[36]^ Current studies on CLICs in HCC lack investigations of epigenetics and the immune microenvironment. In this study, we conducted a bioinformatics analysis of the transcriptional expression, gene mutation, and promoter methylation of CLICs, as well as immune cell infiltration in HCC, to assess the potential usefulness of CLICs as target molecules and prognostic biomarkers.

To identify the transcriptional expression and prognostic value of CLICs in HCC, we investigated the transcriptional expression of CLICs and the correlation between the expression level and pathological stage of HCC. The expression of CLIC members was found to differ between cancer and normal tissues. Consistent with previous research results, we observed that the expression of CLIC1, CLIC3, and CLIC5 was increased in cancer tissues; the expression of CLIC1 and CLIC3 was associated with the progression of the tumor; and a lower expression of CLIC1 was associated with better OS in HCC. We found that the expression of CLIC4 was increased in cancer tissues; however, the mRNA expression of CLIC2 and CLIC6 was lower in HCC tissues than in non-cancer tissues. The expression of CLIC2, CLIC4, CLIC5 and CLIC6 were not associated with the progression of the tumor, whereas the expression of CLIC2–6 were not associated with OS in HCC.

Subsequently, we conducted a network analysis of the 20 functionally related genes of CLICs, which are mostly glutathione S-transferases owing to the structural similarity of CLICs to the glutathione S-transferase family of proteins. We also performed functional enrichment analysis and found that the metabolism of xenobiotics by cytochrome P450, chemical carcinogenesis, and drug metabolism were primarily related to these functions. In previous studies, some post-translational modifications of the CLIC proteins were found or predicted, including ubiquitylation, palmitoylation, phosphorylation, myristoylation, and glycosylation.^[37–39]^ Thus, in this study, we attempted to provide novel clues that may reveal the possible molecular mechanisms at the epigenetic level. We found a genetic alteration rate of 31% for CLICs in HCC (18% for CLIC1 alone). The results showed that the presence of alterations in CLIC1 was significantly correlated with OS in HCC patients. We also identified the relationship between CLIC expression and DNA methylation using UALCAN. The DNA methylation levels of the CLIC1–3 and CLIC5–6 promoters were significantly lower in HCC tissues than in normal tissues, suggesting that methylation may adjust the expression of these CLICs.

Growing evidence demonstrates that immune cell infiltration can affect tumor progression and recurrence as well as the response to immunotherapy and the clinical outcomes.^[40,41]^ CLIC1 has been shown to affect the innate immune system through its expression in macrophages and DCs and by regulating the phagosome proteolytic activity of macrophages.^[7,42,43]^ In our study, a significant correlation was observed between the expression of CLICs and the infiltration of 6 immune cells (B cells, CD8^+^ T cells, CD4^+^ T cells, neutrophils, macrophages, and DCs), indicating that the immune status in HCC may be reflected by CLICs.

We systematically analyzed the expression and prognostic value of CLICs to provide novel clues that may reveal the enigmatic mechanism by analyzing the correlations of CLIC expression with genetic alterations, promoter DNA methylation, and immune cell infiltration. However, our study also had several limitations as all analyzed data were retrieved from online databases and the mRNA levels are not perfect predictors of protein expression.^[44]^ Further cell experiments and clinical sample analyses are needed to confirm our findings and explore the applications of the CLIC members for HCC treatment.

## 5 Conclusion

Our results indicated that the increased mRNA expression and decreased promoter DNA methylation levels of CLICs may play a crucial role in the tumorigenesis of HCC. In addition, the expression of CLIC members was significantly correlated with tumor immune status. High expression levels of CLIC1 and CLIC3 could serve as biomarkers for identifying advanced stages of HCC. Moreover, an 18% mutation rate was also observed for CLIC1, and genetic alterations of CLIC1 were significantly associated with a lower OS in patients with HCC.

## Acknowledgments

The corresponding author thanks his son, Feng Zhu. All authors thank Editage (www.editage.cn) for English language editing.

## Author contributions

**Conceptualization:** Wei Zhu.

**Investigation**: Juan-Jun Huang, Wei Zhu.

**Methodology:** Wei Zhu, Juan-Jun Huang.

**Funding acquisition:** Xiaoli Chen.

**Project administration:** Jing Lin, Wei Zhu.

**Writing – original draft:** Juan-Jun Huang, Wei Zhu.

## Supplementary Material

SUPPLEMENTARY MATERIAL
