# Supplementary material for: Identification of chloride intracellular channels as prognostic factors correlated with immune infiltration in hepatocellular carcinoma using bioinformatics analysis
Source: Medicine (Baltimore). 2021 Nov 12;100(45):e27739. doi: 10.1097/MD.0000000000027739 (PMC10545300; doi:10.1097/MD.0000000000027739)

**Supplemental Figure S1. The correlation between overall survival and immune cell infiltration among patients stratified by CLICs expression level in HCC(TIMER).**

HCC=hepatocellular carcinoma, CLIC=Chloride intracellular channel, HR=hazard ratio.


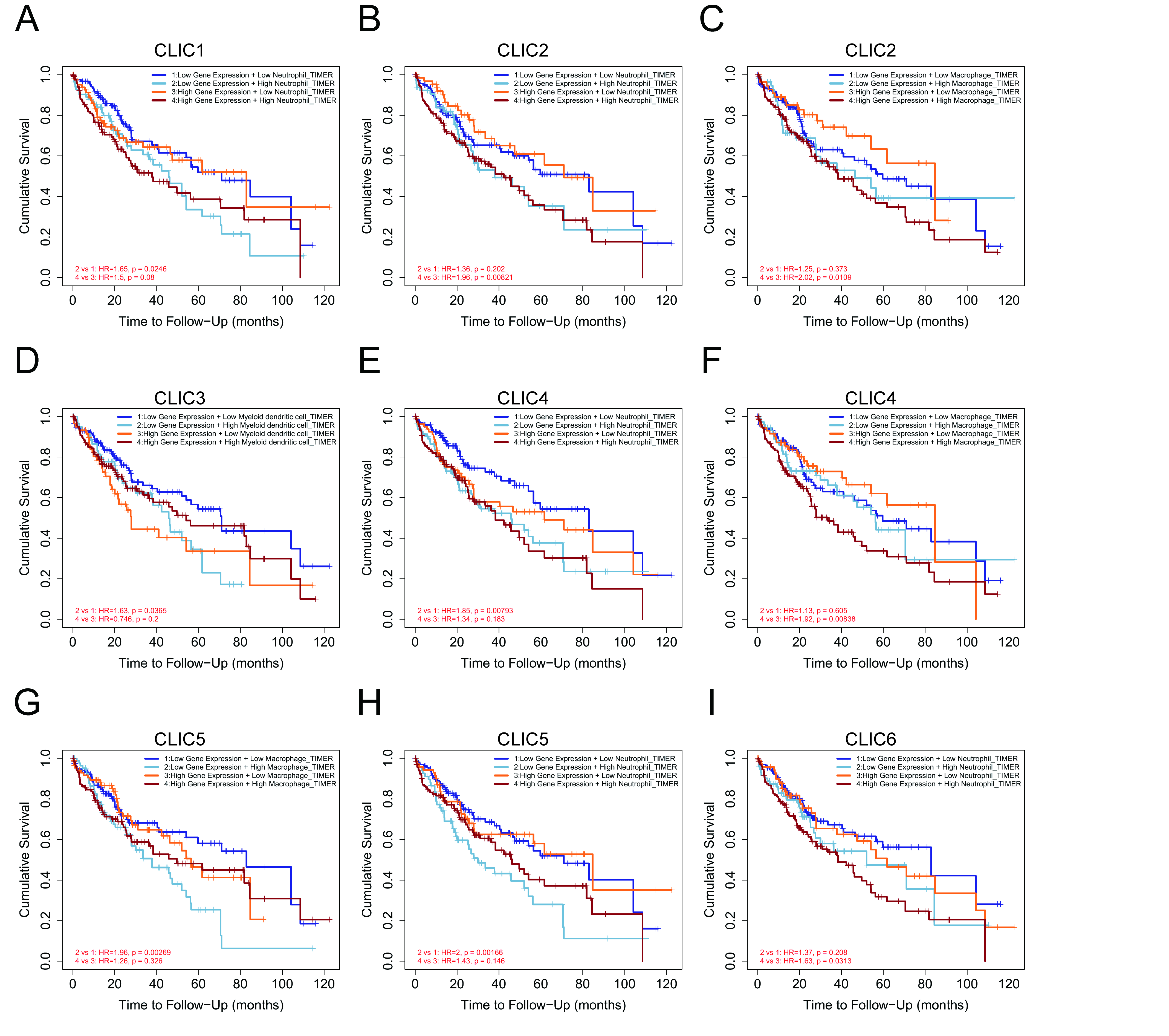

Supplement: SUPPLEMENTARY MATERIAL [file medi-100-e27739-s001.docx]
